# Continuous monitoring of critically ill patients using photoplethysmography—the road to a less invasive ICU monitoring

**DOI:** 10.3389/fdgth.2025.1605020

**Published:** 2025-05-30

**Authors:** João Rosinhas, Rui Malheiro, João Tiago Pimenta, Ricardo Sá, Francisco Serdoura, José-Artur Paiva

**Affiliations:** ^1^ Serviço de Medicina Intensiva, Unidade Local de Saúde São João, Porto, Portugal; ^2^ Departamento de Medicina, Faculdade de Medicina da Universidade do Porto, Porto, Portugal; ^3^ 4LifeLAB, Laboratório Colaborativo, Porto, Portugal; ^4^ EPIUnit—Instituto de Saúde Pública, Universidade do Porto, Porto, Portugal; ^5^ Unidade de Saúde Pública São João, Unidade Local de São João, Porto, Portugal; ^6^ Departamento de Ciências da Saúde Pública e Forenses, e Educação Médica, Faculdade de Medicina da Universidade do Porto, Porto, Portugal; ^7^ Serviço de Cirurgia Geral, Unidade Local de Saúde São João, Porto, Portugal; ^8^ Departamento de Cirurgia e Fisiologia, Faculdade de Medicina da Universidade do Porto, Porto, Portugal; ^9^ Serviço de Ortopedia e Traumatologia, Unidade Local de Saúde São João, Porto, Portugal

**Keywords:** ICU, monitoring, non-invasive, tele-ICU, pulse

## Abstract

**Introduction:**

Intensive Care Medicine is based on continuous timely monitoring of physiological variables to guide modulation of therapy. This monitoring is often invasive, but there is a trend for the adoption of non-invasive devices, already largely used in wards and homecare, to reduce risk of device-associated side effects. The aim of this study was to assess the accuracy of a non-invasive equipment (Corsano Cardiowatch 287-2B) in the assessment of blood pressure, heart rate, temperature and oxygen saturation in critically ill patients admitted to the ICU.

**Method:**

This prospective cohort study developed in an adult ICU admitting patients for level 3 and 2 of care compared the Corsano Cardiowatch 287-2B with the ICU standard monitoring, namely continuous electrocardiogram, invasive arterial blood pressure through arterial catheter, pulse oximeter and central thermometer. Concordance was assessed using the Bland-Altman test.

**Results:**

Nineteen patients were included in the study. The number of time-points included for comparison between the two monitoring strategies were more than 50,000 in pulse and heart rate, around 40,000 in oxygen saturation and body temperature and 1,200 in systolic and diastolic blood pressure. Bias for heart rate and pulse were −1.73 and −0.77, respectively. The limits of agreement were between −14.90 and 11.33, for heart rate, and −14.25 and 12.71, for pulse. Small biases were also estimated for oxygen saturation (0.21), with limits of agreement between −6.97 and 7.39, and body temperature (0.58), with limits between −1.12 and 2.47. Concordance was low for diastolic and systolic blood pressure, with bias of 5.18 and −11.27, respectively.

**Conclusions:**

Corsano Cardiowatch 287-2B reaches good levels of concordance compared to traditional ICU monitoring for heart and pulse rates and may be a valuable solution for their less invasive monitoring, with promising results for future operationalization for oxygen saturation and body temperature. Concordance is low for blood pressure, meaning the device is currently unsuitable for use with that purpose.

## Introduction

Intensive Care Medicine is based on continuous timely monitoring of vital signs and physiological variables to guide modulation of therapy and care. Continuous advanced cardiorespiratory monitoring is mainly restricted to intensive care units (ICU), operating rooms, and post-anesthesia care units. Currently, most advanced cardiorespiratory monitoring systems still depend on invasive sensors, such as arterial and central venous lines (1). However, there is a clear and solid trend for the adoption and use of non-invasive devices to obtain monitoring of those relevant variables, with similar validity and improved feasibility, while increasing patient safety by avoiding the risk of endovascular device-associated side effects (2, 3). Invasive monitoring methods are sometimes associated with meaningful complications, such as thrombosis, pseudoaneurysms, bloodstream infections, neuronal lesions, hematomas or bleeding, in the case of arterial blood lines (4), or esophageal lesions and hemorrhage, in the case of esophageal temperature probes (5).

This non-invasive monitoring has been largely applied in the wards and in home care often with telemetric communication to emergency systems (6). However, it is often basic and always intermittent. Automated continuous noninvasive ward monitoring is a promising approach to closely follow changes in vital signs over time and thus identify patients who are deteriorating in a timely fashion (7). There is evidence that most patients show signs of physiological deterioration in the 12 h before an intra-hospital cardiac arrest (8) and early recognition of the clinical deterioration that anticipates progressive organ dysfunction allows an early encounter of this patient with an Intensive Care Team that often impacts on outcome (9).

Even in the ICU, the use of non-invasive devices has become standard of care, namely pulse oximetry, transcutaneous oxygen and carbon dioxide measurement devices (for assessment of arterial oxygen saturation and peripheral perfusion), near infrared spectroscopy (for cerebral oxygenation) and electroencephalography (for brain activity). The broader use of those devices reduces the use of invasive catheters and sensors, and therefore their associated complications, and the drawing of blood samples. However, non-invasive devices, namely hemodynamic non-invasive devices, are prone to artifacts and may lack the precision of invasive methods in certain clinical scenarios, such as in patients with hemodynamic instability, severe hypoxia, or multi-organ dysfunction or even in patients with arrhythmias or low cardiac output (10).

Although there is a research trend on wearable devices for non-invasive monitoring, most have either focused on a single outcome (11–14) or has addressed a specific population (15–18). To the best of our knowledge, only one study assessed a chest patch in ICU patients to test its reliability for cardiac output measurements (11). Therefore, the potential of these devices for all relevant cardiovascular measurements is yet to be established in this population.

The aim of this study was to assess the accuracy of a non-invasive equipment in the assessment of blood pressure, heart rate, temperature and oxygen saturation in critically ill patients admitted to an ICU and comparing it with the ICU standard monitoring, namely 4-points continuous electrocardiogram, invasive arterial blood pressure through arterial catheter, pulse oximeter and central thermometer.

## Methods and population

This was a prospective cohort study developed in an adult ICU admitting patients for level 3 and 2 of care of an Intensive Care Department in a university hospital.

We included all consecutive adult patients admitted to the ICU fulfilling the inclusion criteria and without any of the exclusion criteria. Inclusion criteria were admission to the ICU with standard multimodal basal monitoring, namely the concomitant monitoring of arterial blood pressure through arterial catheter sensor, pulse plethysmography and oximetry to obtain peripheral oxygen saturation, and pulse rate and tympanic or esophageal sensor for body temperature. Exclusion criteria were: (a) one of two signs of inadequate peripheral perfusion, either mottling score >2 or capillary refill time >2 s; (b) difference of digital sphygmomanometer acquired systolic blood pressure measurement above 20 mmHg between the two arms; (c) invasive blood pressure sensor through a femoral artery catheter; (d) existence of refractory shock defined as serum lactate >4.0 mmol/L or norepinephrine perfusion >0.5 mcg/Kg/min; (e) body temperature below 34°C; (f) patient on extracorporeal circulation; (g) amputation of at least one of the superior limbs.

The Unidade Local de Saúde São João's Ethical Committee approved the study protocol (approval number 80/2024). To be enrolled, the patients had to accept to be part of the study via informed consent. For patients who were not capable of taking that decision, the informed consent was given by their legal representative. We performed the trial in accordance with the principles of the Declaration of Helsinki. The enrollment period was July and August 2024.

The data was collected by a wearable photoplethysmography device named Corsano Cardiowatch 287-2B (CW2). The bracelet was used in the wrist opposite the arterial line. All the parameters were then constantly transferred, via Bluetooth®, to a dedicated cellphone with the Corsano's app. There was no human manipulation of data and no display on the watch. This application worked as an intermediator between the acquisition of data from the wearable and Corsano's trial online database cloud. Every patient was pseudonymized with an untraceable alphanumeric code, the data was properly encrypted and only accessible by the investigation team. When data collection was terminated, all the data and information was deleted form the wearable and cellphone, ensuring that there were no leaks in information.

The heart rate, pulse, body temperature, peripheral oxygen saturation and blood pressure levels were all measured directly in the wearable, rechargeable device. This device utilizes photoplethysmography (PPG) to perform measurements, combining signals from light sources, light sensors, electrodes, and an accelerometer. Multiple features from those signals are interpreted by an artificial intelligence algorithmic model (11), allowing continuous evaluation of the vital signs. For calibration, blood pressure needs three sets of three measurements, at three different times of the day, with a classical sphygmomanometer which was also connected via Bluetooth®. CW2 is capable of measuring blood pressure every 30 min and all the other variables every minute. The promoter of this study—4LifeLab—was responsible for acquiring the device and the cloud for data storage from Corsano, as well as for recruiting the researchers. Corsano had no interference, direct or indirect, in the development and eventual publication of our research.

All the above parameters were compared with the gold standard bedside modules of Philips IntelliVue MX550® or MP70® multimodal continuous monitors. For this standard methodology, temperature was obtained using MedLinket W0003F® esophageal probes, arterial blood pressure information was gathered with BBraun Combitrans® Monitoring Kit system and Philips M1191BL® or Masimo RD SET™ DCI® systems were used for measuring peripheral oxygen saturation. Both the gold standard blood pressure device and the cardiowatch required calibration for blood pressure prior to their use.

The wearable device only needed to be removed for surgery and magnetic resonance, being completely compatible with all other moments, namely bath. The nursing team was properly educated for the correct usage and cleaning of the wearable and peripherals. CW2 measures the quality of the signal by quantitative and percentual method. Missing and poor-quality signal (below 80% signal quality) values, either in the standard or in the wearable device, were excluded. As the CW2 temperature sensor was designed as a fever detector and monitor, being calibrated only for measurements above 36,5°C, all the measurements below 36,5°C were disregarded.

### Variables

For each included patient, heart rate (in beats per minute), pulse (in beats per minute), body temperature (in degrees Celsius), oxygen saturation levels (in percentage) and blood pressure levels (in mmHg) were retrieved simultaneously by both the Corsano Cardiowatch 287-2B (CW2) and the gold standard method. Patients were monitored continuously by the gold standard ICU method. Regarding CW2, blood pressure levels were measured every 30 min, whereas the remaining variables were measured once per minute, for 72 h. All the values measured by the standard method viewed by the investigators as “biologically non-plausible”, namely oxygen saturations below 80% and temperatures below 35°C, were disregarded to avoid potential measurement bias.

Demographic variables (age and sex), admission type (neurocritical, medical, surgical, polytrauma), patient adult critical care level, the presence of invasive mechanical ventilation, existence or not of deep sedation, vasopressor support and obesity (defined as a body mass index above 30 kg/m^2^), were retrieved for each patient and used for the description of the population. The APACHE II score and the SAPS II score, both estimating hospital mortality upon admission to intensive care units, were also retrieved.

### Statistical analysis

Descriptive data were reported as absolute and relative frequency, and medians with interquartile range, where applicable. The parameters retrieved by CW2 and the gold standard method were matched, per patient on a per-minute basis.

Concordance of estimates was assessed using the Bland-Altman test for the entire sample, for each parameter included. The mean difference between the two methods of measurement is given by bias. A significance level of 0.05 was considered for the estimation of the limits of agreement, which enclose the range in which 95% of the observed differences between methods lie. The density distributions were plotted to visually compare the distributions of both methods. A sensitivity analysis was performed, stratifying patients into the categories used for the descriptive analysis, to assess whether the performance of the bracelet was different for subsets of the population.

Analysis was performed using software R, version 4.4.1, using the ‘blandr’ package.

## Results

A total of 19 patients were included in the study. Baseline characteristics are provided in Table 1. Two patients had no estimates of body temperature, whereas the comparisons in blood pressure included 8 patients only. Most patients were males (68%), with a median age of 65. Patients under invasive mechanical ventilation (74%) were the same patients classified as being under deep sedation, under vasopressor support and at level 3 of Adult Critical Care. For oxygen saturation, 42 897 out of 42 910 measurements (99.7%) were kept after excluding biologically non-plausible measurements in the gold-standard. For body temperature, 38 147 out of 42 290 (88.1%) were retained.

**Table 1:** Baseline characteristics of patients.

Variables	No. (%)
Age (median, IQR)	65.0 (54.5–78.0)
Male sex	13 (68)
Admission type	
Medical	9 (47)
Surgical	6 (32)
Polytrauma	3 (16)
Neurocritical	1 (5)
APACHE II score at admission (median, IQR)	24.0 (18.5–27.5)
SAPS II at admission (median, IQR)	51.0 (38.5–67.0)
Invasive mechanical ventilation	14 (74)
Deep sedation	14 (74)
Obese	6 (32)
Vasopressor support	14 (74)
Adult critical care level	
Level II	5 (26)
Level III	14 (74)

IQR, interquartile range.

**Figure 1:**
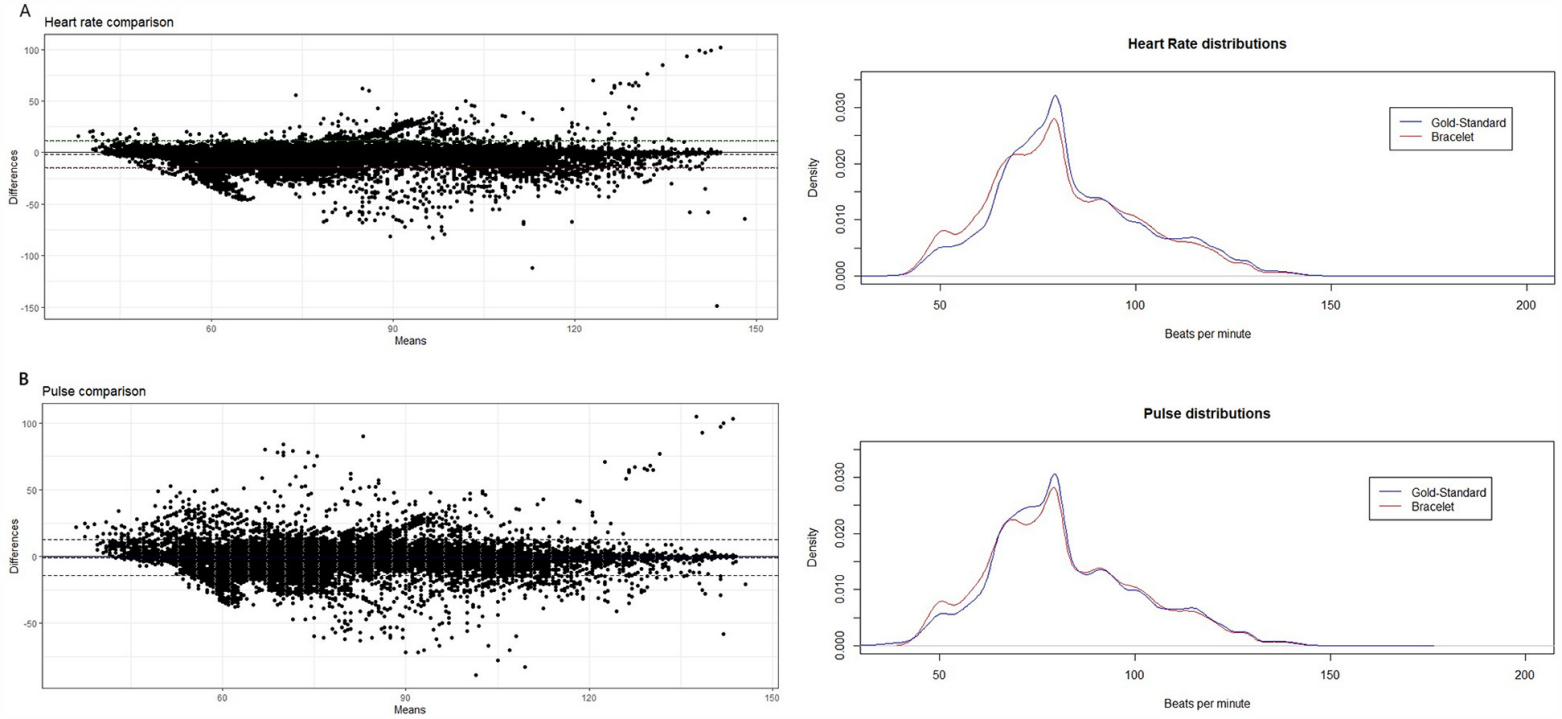
Bland–Altman plots of agreement and density distributions of **(A)** heart rate and **(B)** pulse rate.

Bias for heart rate and pulse were −1.73 and −0.77, respectively, with over 3 500 mean comparisons per patient. The limits of agreement were between −14.90 and 11.33, for heart rate, and −14.25 and 12.71, for pulse. The graphical display of the analysis and density distributions are shown in Figure 1, suggesting the approximate same pattern for both traditional monitoring and CW2 for these variables.

**Figure 2:**
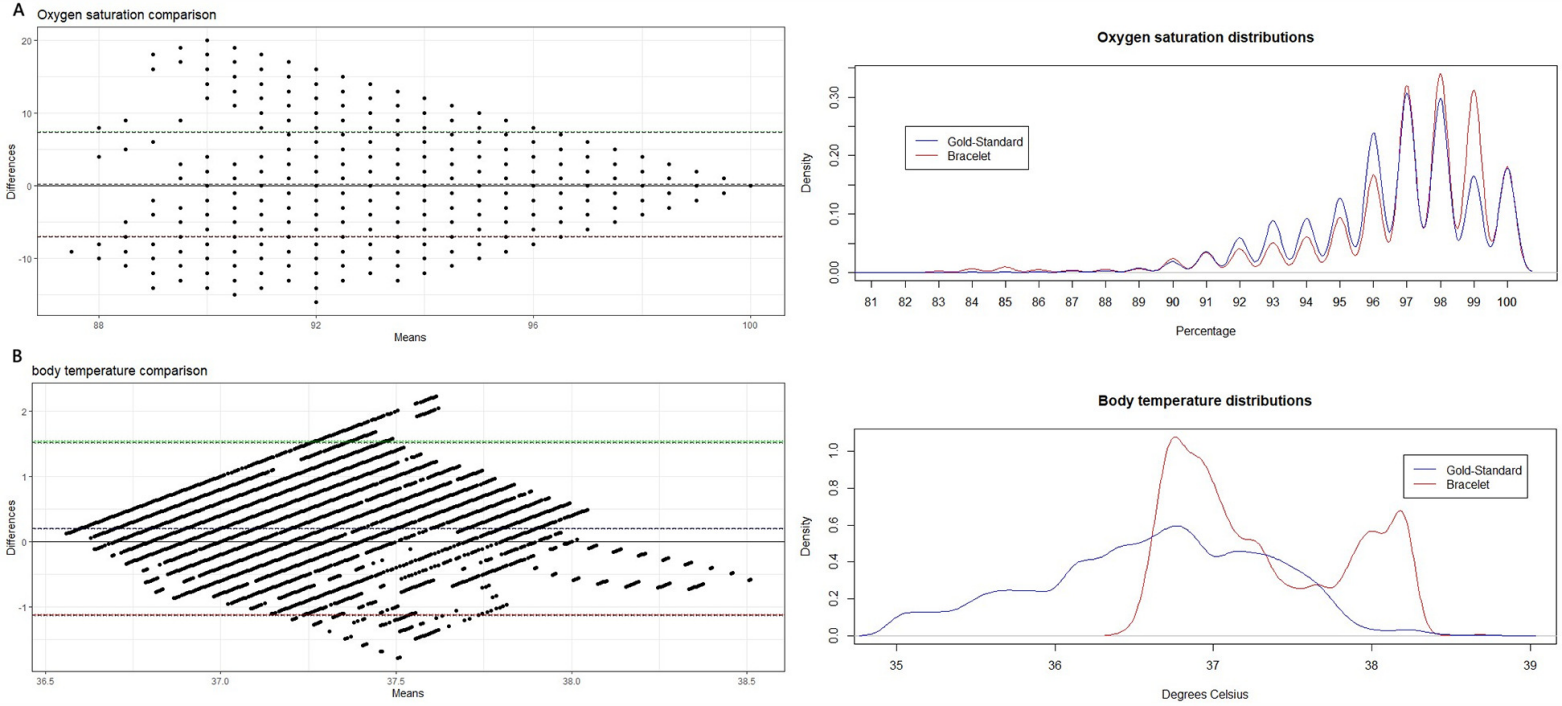
Bland–Altman plots of agreement and density distributions of **(A)** oxygen saturation and **(B)** body temperature.

Small biases were also estimated for oxygen saturation (0.21), with limits of agreement between −6.97 and 7.39, and body temperature (0.58), with limits between −1.12 and 2.47 (Figure 2).

Diastolic and systolic blood pressure were evaluated separately, and the number of comparisons was far below that obtained for other variables (1200). Bias was 5.18 and −11.27, respectively (Supplementary Figure S1). Detailed data on all variables is available on Table 2.

**Table 2:** Summary results, by physiological variable.

Variable	Unit	Mean no. comparisons	Total no comparisons	Bias	Limits of agreement
Heart rate	Beats per minute	3,505	56,467	−1,7	[−14.90 to 11.33]
Pulse	Beats per minute	3,510	53,841	−0,8	[−14.25 to 12.71]
Oxygen saturation	%	2,018	42,897	0,2	[−6.97 to 7.39]
Body temperature	°C	2,235	38,147	0,6	[−1.12 to 2.47]
Diastolic blood pressure	mmHg	49	1,200	5,2	[−25.68 to 26.04]
Systolic blood pressure	mmHg	49	1,200	−11,3	[−61.42 to 38.88]

The sensitivity analysis showed no major effect of any descriptor in the estimates (Supplementary Table S1).

## Discussion

Our study shows that CW2 reaches good levels of concordance compared to traditional ICU monitoring for heart and pulse rates, and estimates are promising for oxygen saturation and body temperature, although they may require further precision adjustments. Concordance is low for blood pressure, meaning the device is currently unsuitable for use with that purpose.

Although limits of agreement for heart and pulse rates may be slightly wide, meaning that occasionally one could over or underestimate true values by up to 10 bpm, the plotted cloud and mean bias suggest that CW2 is very reliable for the monitoring of these variables. There seems to be no pattern of deviation in the plot, and thus differences in concordance may relate to random variation. Those results are aligned with previous trials developed by Monnink et al. and Blok et al. to test CW2 accuracy for heart or pulse rates in hospitalized patients (18, 19). Good results were also obtained in cardiovascular non-hospitalized patients with the same (16, 20, 21) and other devices (17, 22).

Regarding peripheral oxygen saturation (spO2), our study shows an almost negligible bias (0.21%) but suboptimal limits of agreement. These limits are sufficient to limit the usefulness in clinical practice at the moment, as one may not miss a saturation level by 6 or 7%. However, the existence of measurement errors on the gold standard arm is possible, as it provided many values between 80% and 88%, which are rare and ephemeral in the ICU. In fact, pulse oximeter sensors used for spO2 measurement in the ICU are very dependent on the peripheral perfusion and light in the surroundings. In real-life, nurses and doctors tend to disregard values that are numerically low but have suboptimal spO2 wave and promptly correct the sensor placement. As the values were collected every minute, we suspect that most of the spO2 < 88% were wrongly obtained by the standard method, but since we have only the numerical value on our database, and not the corresponding curves, we had no grounds to disregard those values. Nevertheless, the inclusion of those values is highly relevant since patients with low oxygen saturation are particularly critical and represent those patients and moments that monitoring devices must not miss. The distribution of the CW2 values suggests that it may be missing primarily patients with 99% saturation and, thus, optimization may require keener attention on that subset. Results in spO2 are comparable to other studies, testing different devices in healthy populations (12, 14), strengthening the potential external validity of our study. In 2024, two studies were published testing CW2 accuracy in spO2 measurements with more optimistic results. One by Vijgeboom et al. (limits of agreement −2,37 and 2,62) and other lead by Monnink (limits of agreement −2,3 and 3,37) (16, 18). It is worth mentioning that the first study was developed in healthy individuals during high-intensity interval training and the second in cardiac patients in the setting of elective heart catheterization.

Concerning body temperature, even though the mean bias was only 0.58°C, the limits of agreement were also suboptimal. Missing a temperature measure by 1 or 2°C is sufficient to miss an episode of fever when presented, or to identify fever in a normopyretic patient. Furthermore, the fact that the CW2 temperature sensor was designed as a fever detector and is calibrated only for measurements above 36.5°C makes it unsuitable for temperature monitoring of critically ill patients. Having said that, the distribution of temperature values obtained with CW2 is rather plausible for a critical care population and, on the other hand, the distribution of the values obtained by gold standard ICU monitoring shows many measurements below 36.5°C and very few fever events. The standard temperature measurements were taken with esophageal probes who are prone to dislodgment and exteriorization, and the degree of exteriorization is inversely proportional to the temperature value. A second study with more robust gold standard data and a new algorithm capable of measuring values above 35.5°C will be performed in the near future. We also argue that the most relevant part of the comparison is that both tools may effectively distinguish between patients with normal, supranormal or subnormal levels of temperature. That analysis should also be a part of the future study. Other studies assessed the validity of wearables (patches) for body temperature measurement, albeit for different populations, and found a high concordance, suggesting that it may be possible to recalibrate the Corsano CW for practical use for this outcome (23–25).

For blood pressure (BP) measurements, the bias is extremely large. Although the number of comparisons is markedly smaller than for other variables, they were enough to have find concordance if it existed. Unsatisfactory results in blood pressure are aligned with previous studies, with different devices (13, 26). It is important to note that our results are rather different from those obtained by van Vliet et al. in a previous study comparing CW2 vs. arterial line blood pressure measurements (27). In that study the mean errors were ±3.7 mmHg (SD 4.4 mmHg) and ±2.5 mmHg (SD 3.7 mmHg) for systolic and diastolic BP, respectively. The population and methodology of both studies could justify those differences. In the van Vliet et al. study, patients were ambulatory and not in a critical care unit, time of monitoring was 4 min and not 72 h and even the site of arterial line placement was different (subclavian artery vs. radial/brachial artery). Moreover, our study had almost triple the amount of comparison points and was developed in a more “real-life” and continuous manner. The downside of a more realistic setting is that it is not uncommon to have low quality arterial line measurements either by over or underdamping phenomena, patient movements, transient peripheral arterial vasospasm or pressor sensor displacement. We speculate that those common measuring errors could produce measurements that are within the “biological plausible values”, and therefore not excluded from the study, but nonetheless incorrect. A second study will be developed under a 19-bit Analog-to-Digital Converter, that could potentially augment processing precision, improving the results obtained with the CW2.

Our study has several limitations. First, it is a monocentric study. Secondly, only 19 patients were included. However, the total number of time-points included for comparison between the two monitoring strategies were more than 50,000 comparisons in pulse and heart rate, around 40,000 comparisons in oxygen saturation and body temperature and 1,200 comparisons in systolic and diastolic blood pressure. Therefore, the internal validation of the paper is high—many measurements per patient—but the external validation needs to be confirmed and is only applicable to a similar population—critically ill patients on level 2 or 3 of care. The device was not tested in highly unstable patients and results must be handled with care in that population. As discussed, the gold-standard measurements are often prone to errors and artifacts, which, by assessing concordance, may have underestimated the accuracy of CW2. The fact that ICU patients tend to be more similar between hospitals than other subgroups of patients and that their mobility is tendentially reduced may favor the external validity of the results. Moreover, the absence of an effect in the sensitivity analysis supports the notion that some characteristics of this population may not significantly impact the performance of CW2.

Despite a significant number of trials studying the usability of non-invasive methods of continuous vital sign monitoring, to the best of our knowledge, our study is unique in studying such a severely ill population with a very high number of points of comparison.

## Conclusion

Corsano Cardiowatch 287-2B may be a valuable solution for less invasive monitoring of heart rate and pulse levels, with promising results for future operationalization for oxygen saturation and body temperature. Further developments are needed for adequate monitoring of the full array of variables monitored in critically ill patients.

## Data Availability

The raw data supporting the conclusions of this article will be made available by the authors, without undue reservation.
